# Role of the *PRNP* S127 allele in experimental infection of goats with classical caprine scrapie

**DOI:** 10.1111/age.12291

**Published:** 2015-04-27

**Authors:** Rohana P. Dassanayake, Stephen N. White, Sally A. Madsen‐Bouterse, David A. Schneider, Katherine I. O'Rourke

**Affiliations:** ^1^ Department of Veterinary Microbiology and PathologyCollege of Veterinary MedicineWashington State UniversityPullmanWA99164‐6630USA; ^2^ Animal Disease Research UnitAgricultural Research ServiceU.S. Department of AgriculturePullmanWA99164‐6630USA

## Background

Classical scrapie is a transmissible spongiform encephalopathy affecting domestic goats and sheep and associated with accumulation of a misfolded isoform (PrP^Sc^) of the *PRNP* gene product. Multiple *PRNP* polymorphisms have been reported in goats.^1^, ^2^ Experimental scrapie inoculation studies revealed that *PRNP* polymorphisms at codons 146, 154, 211, and 222 can provide resistance or a prolonged incubation period.^3^, ^4^, ^5^ A recent study identified the association between a polymorphism at codon 127 (G127S) and reduced probability of developing clinical signs of scrapie in goats with PrP^Sc^ detectable in the brain.^6^ In that study, the length of the incubation period (time from infection to clinical signs) was not known. The aim of this study was to identify whether goats with the heterozygous (G/S_127_) genotype have an extended incubation period compared with goats homozygous for G_127_ (G/G_127_) following classical caprine scrapie inoculation.

## Methods

Intracerebral inoculation was performed with brain homogenates from two natural field cases of scrapie, one G/G_127_ and one G/S_127_, as described (Table S1). Data were combined from previous reports^4^, ^5^ to achieve efficient animal use. Because the previous report^6^ suggested a prolonged incubation time for G/S_127_ goats, a one‐tailed nonparametric exact test (see Appendix S1) was used to assess the recipient genotype hypothesis that G/S_127_ goats have longer incubation times than do G/G_127_ goats when intracerebrally inoculated with classical caprine scrapie from G/G_127_ genotype source material. In the absence of preliminary data, two‐tailed nonparametric exact tests were used for all other analyses.

## Results/Discussion

All inoculated goats developed clinical signs of scrapie (Table S1), with disease confirmed by the detection of PrP^Sc^ in brainstem at the obex and most lymphoid tissues by immunohistochemistry^8^ (Fig. S1). A significant (*P *=* *0.019) increase in incubation time was observed in G/S_127_ goats following inoculation with the G/G_127_ caprine brain homogenate (range: 647–1333 days). No significant difference in incubation time was observed for G/G_127_ versus G/S_127_ inoculum in G/G_127_ goats (*P *=* *0.30). Incubation times in control G/G_127_ goats inoculated with the G/G_127_ brain homogenate (range: 261–332 days) were significantly shorter than observed in prior work (both *P *< 0.05^4^, ^5^). Our findings include the first reported classical scrapie incubation times for G/S_127_ goats, confirming that S127 is not protective in goats.^6^, ^7^ These data suggest that eradication programs need to include extended traceback periods and longer post‐exposure monitoring times for infected herds containing G/S_127_ goats.

## Supporting information


**Appendix S1** Supplementary Methods.

**Figure S1** Detection of PrPSc immunolabeling in lymph nodes and brainstem at the obex of G/S127 goats intracerebrally inoculated with classical caprine scrapie.

**Table S1** Intracerebral inoculation of goat kids with homozygous (G/G127) or heterozygous (G/S127) brain‐derived inocula prepared from clinically affected goats with naturally acquired classical caprine scrapie.
