# Supplementary material for: Role of the PRNP S127 allele in experimental infection of goats with classical caprine scrapie
Source: Anim Genet. 2015 Apr 27;46(3):341. doi: 10.1111/age.12291 (PMC5132141; doi:10.1111/age.12291)
Supplement: Supplementary file 1 — Appendix S1 Supplementary Methods. [file AGE-46-341-s001.pdf]

Appendix S1. Supplementary Methods

Saanen kids from a scrapie-negative herd at Washington State University, Pullman WA USA were transferred to clean outdoor pens at age six to seven months. Kids were anaesthetized and intracerebrally inoculated with 1 mL of 10% (w/v) brain homogenate prepared from clinically affected goats with naturally acquired classical caprine scrapie and either G/G127 or G/S127 inoculum source gentoype (Table S1). One age-matched G/G127 kid was raised with the inoculated goats as an uninoculated control. Animals were monitored daily and humanely euthanized once advanced signs of clinical scrapie developed (Table S1). The Washington State University Institutional Animal Care and Use Committee approved all procedures (ASAF 3811, 3815 and 4107). Brain, alimentary tract-associated lymphoid tissues (retropharyngeal, tonsils, mesenteric, ileocecal junction, ileum, and spleen), and peripheral lymph nodes (prescapular, prefemoral, and popliteal) were collected at necropsy and evaluated for PrPSc accumulation by scrapie immunohistochemistry as previously described (1) using tissues from the uninoculated goat as negative controls.

Statistical analysis was performed using the npar1way procedure of SAS 9.2 (SAS Institute, Cary, NC). Specifically, exact nonparametric Wilcoxon rank-sum tests were used to test all hypotheses. Exact tests provided correct handling of small sample sizes. In the case of the 2recipient genotype hypothesis (G/G127 inoculum into either G/G127 or G/S127 recipients), preliminary data suggested G/S127 heterozygotes would have increased incubation times (2), so a one-tailed test was used to assess this hypothesis. For all other hypotheses, no preliminary data existed so a two-tailed test was used. To be globally representative and conservative in declaring a significant difference in incubation time, we used data from all published reports of

intracerebral goat challenge with genotype-defined classical caprine scrapie inoculum into genotype-defined goat recipients as part of the control group (G/G127 inoculum and G/G127 recipients) for analysis of both hypotheses (3,4). This is conservative in that previous reports of G/G127 source material intracerebrally inoculated into G/G127 recipients showed somewhat longer incubation times (Table S1; 3,4), this should result in a mean control incubation time closer to the means of both the G/S127 heterozygotes (recipient genotype hypothesis) and G/G127 recipients with G/S127 inoculum (inoculum genotype hypothesis), resulting in a more stringent threshold for declaring significance at $P<0.05$.

References:

1. O'Rourke K.I. et al. (2012) BMC Vet Res 8, 42. 40

2. Goldmann W. et al. (2011) Vet Res 42, 110. 39

3. Lacroux C. et al. (2014) J Virol 88, 2406-2413. 37

4. Acutis P.L. et al, (2012) Vet Res 43, 8. 38
