# Supplementary material for: Role of the PRNP S127 allele in experimental infection of goats with classical caprine scrapie
Source: Anim Genet. 2015 Apr 27;46(3):341. doi: 10.1111/age.12291 (PMC5132141; doi:10.1111/age.12291)

Figure S1. Detection of PrP^{Sc} immunolabeling in lymph nodes and brainstem at the obex of G/S_{127} goats intracerebrally inoculated with classical caprine scrapie. PrP^{Sc} immunolabeling (dark red) was visible in the lymphoid follicles in the retropharyngeal lymph nodes (a), ileocecal lymph nodes (b), prescapular lymph nodes (c) and dorsal motor nucleus of vagus of the brainstem at the obex (d). IHC was performed using mAb F99/97.6.1 (5 µg/mL) and AEC substrate-chromogen. No immunolabeling was seen in paired tissues from the uninoculated goat housed with the inoculated goats (data not shown).

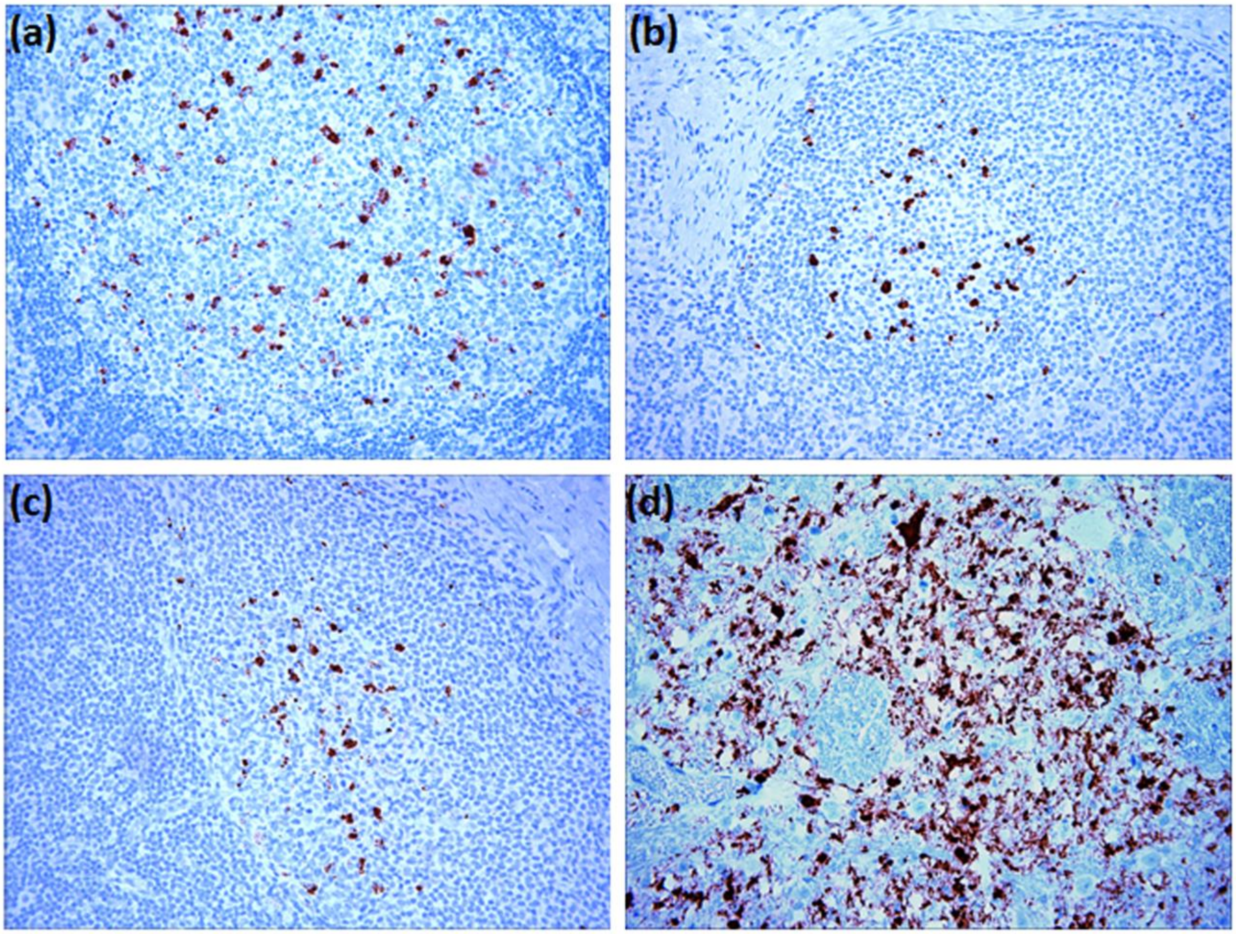

Supplement: Supplementary file 2 — Figure S1 Detection of PrPSc immunolabeling in lymph nodes and brainstem at the obex of G/S127 goats intracerebrally inoculated with classical caprine scrapie. [file AGE-46-341-s002.pdf]
