# Supplementary material for: Role of the PRNP S127 allele in experimental infection of goats with classical caprine scrapie
Source: Anim Genet. 2015 Apr 27;46(3):341. doi: 10.1111/age.12291 (PMC5132141; doi:10.1111/age.12291)
Supplement: Supplementary file 3 — Table S1 Intracerebral inoculation of goat kids with homozygous (G/G127) or heterozygous (G/S127) brain‐derived inocula prepared from clinically affected goats with naturally acquired classical caprine scrapie. [file AGE-46-341-s003.pdf]

Table S1. Intracerebral inoculation of goat kids with homozygous (G/G_{127}) or heterozygous (G/S_{127}) brain-derived inocula prepared from clinically affected goats with naturally acquired classical caprine scrapie.

Donor goats *PRNP* genotype	Recipient kids *PRNP* genotype	Number of clinically scrapie affected animals /total number of animals	Scrapie incubation period[a] (dpi, mean ± SD)	PrP^Sc detection number of positive animals /total number of animals CNS[b]	Lymphoid tissues[c]
Previous studies					
G/G_{127}	G/G_{127}	5/5	486 ± 21[d]	5/5[d]	5/5[d]
G/G_{127}	G/G_{127}	5/5	557 ± 103[e]	5/5[e]	5/5[e]
Present study					
G/G_{127}	G/G_{127}	3/3	289 ± 37	3/3	3/3
G/G_{127}	G/S_{127}	2/2	647, 1333[f]	2/2	2/2
G/S_{127}	G/G_{127}	2/2	343, 358[f]	2/2	2/2

Abbreviations: G = glycine; S = serine; *PRNP* = prion gene; dpi = days post-inoculation; SD = standard deviations; PrP^{Sc} = misfolded isoform of prion protein was detected by immunohistochemistry; [a]interval (days) from inoculation to appearance of clinical signs of scrapie; [b]CNS = central nervous system examined at or immediately posterior to the brainstem at the obex; [c]alimentary tract-associated lymphoid tissues (retropharyngeal, tonsil, mesenteric, ileocecal junction, ileum, and spleen), and peripheral lymph nodes (prescapular, prefemoral, and popliteal); [d] from Lacroux C. *et al* (2014) *J Virol* **88**, 2406-2413; [e]from Acutis P.L. *et al* (2012) Vet Res **43**, 8; [f]for experimental groups with fewer than 3 individuals, actual data values are reported instead of mean ± SD.
